# The efficacy, safety, and immunogenicity of plague vaccines: A systematic literature review

**DOI:** 10.1016/j.crimmu.2023.100072

**Published:** 2023-11-01

**Authors:** Louise Hartley, Sydney Harold, Emma Hawe

**Affiliations:** RTI Health Solutions, The Pavilion, Towers Business Park, Wilmslow Road, Didsbury, Manchester, M20 2LS, UK

**Keywords:** Plague vaccine, *Yersinia pestis*, Bubonic plague, Pneumonic plague

## Abstract

Plague remains endemic in many parts of the world, and despite efforts, no preventative vaccine is available. We performed a systemic review of available randomised controlled trials (RCTs) of live, attenuated, or killed plague vaccines vs. placebo, no intervention, or other plague vaccine to evaluate their efficacy, safety, and immunogenicity. Data sources included MEDLINE, Embase, and the Cochrane Library; clinical trial registers; and reference lists of included studies. Primary outcomes were efficacy, safety, and immunogenicity. Risk of bias was assessed using the Cochrane Collaborations tool. Only 2 RCTs, both on subunit vaccines, were included out of the 75 screened articles. The 2 trials included 240 participants with a follow-up of 3 months and 60 participants with a follow-up of 13 months, respectively. Safety evidence was limited, but both vaccines were well tolerated, with only mild to moderate adverse events. Both vaccines were immunogenic in a dose-dependent manner. However, given the limited data identified in this systematic review, we are unable to quantify the efficacy of vaccines to prevent plague, as well as their long-term safety and immunogenicity. More trials of plague vaccines are needed to generate additional evidence of their long-term effects.

## Highlights

• We systematically reviewed vaccines currently available for plague prevention.
• Evidence from clinical trials was limited, with 2 early phase trials identified.
• Further research is needed to evaluate vaccines for bubonic or pneumonic plague.

## Graphical abstract

**Figure:**
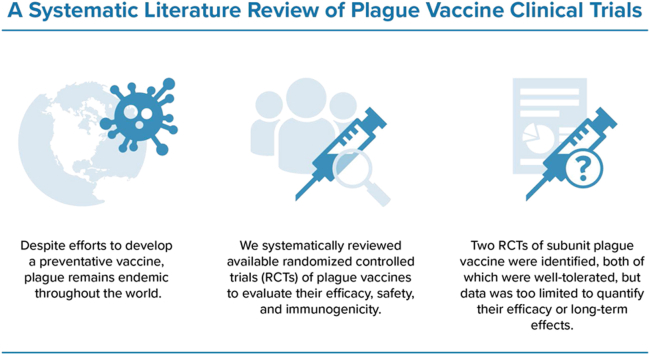

## 1 Introduction

Plague is an infectious disease caused by the *Yersinia pestis* bacterium (Barbieri et al., 2020). It is still endemic in many countries throughout the world where the risk of human plague is increased due to the occurrence (World Health Organization, 2022) of the following: plague bacterium, an animal reservoir, a vector, and the human population. Plague is usually found in three main forms: bubonic, septicaemic, and pneumonic (Dennis et al., 1999). Bubonic plague is transmitted through flea bites and direct contact with an infected animal. Symptoms of bubonic plague include fever, the swelling of lymph nodes, headache, and weakness. Septicaemic plague occurs when plague bacteria directly enter the bloodstream and multiply. Symptoms include weakness, nausea, vomiting, diarrhoea, and the skin turning black. Septicaemic plague can be a complication of both bubonic and pneumonic plague or can occur independently (Centers for Disease Control and Prevention, 2018). Unlike bubonic and septicaemic plague, pneumonic plague is transmitted from person to person by infectious droplets. This type of plague occurs when plague bacteria infect the lungs either through direct inhalation or through the secondary spread of bacteria from bubonic plague or septicaemia. Symptoms of pneumonic plague include shortness of breath, cough, chest pain, and bloody sputum.

Cases of all forms of plague are still reported worldwide, with plague being endemic in countries such as China, Vietnam, and India, and in large parts of Southern Africa (World Health Organization, 2022; Shi et al., 2018; Respicio-Kingry et al., 2016; Stenseth et al., 2008; Bertherat, 2019). From 2010 to 2015, approximately 3248 cases were reported (Bertherat, 2016). Furthermore, there was a large outbreak of plague in Madagascar in 2017. In this outbreak, there were 1791 cases of pneumonic plague, 1 case of septicaemic plague, and 314 cases of bubonic plague. Unspecified plague was reported in 215 cases (World Health Organization, 2017). Especially troubling is that the 2017 outbreak in Madagascar demonstrated several atypical characteristics, occurring outside the usual seasonal timeframe (August to November, rather than October to April), in an urban area, and with a high ratio of pneumonic to bubonic cases (World Health Organization, 2018).

Although vaccination is thought to be an efficient way to protect against plague in the long term, there is currently no approved licensed vaccine available for plague, and the World Health Organisation (WHO) does not recommend immunisation with old generation plague vaccines (World Health Organization, 2018; Sun and Singh, 2019). Evidence for the efficacy and safety of other plague vaccines is scarce. A 1998 Cochrane review found no randomised controlled trials (RCTs) had been conducted to evaluate the efficacy of any plague vaccine (Jefferson et al., 2000). This review has since been updated in 2006, 2009, and 2011, but no new studies were identified (Jefferson et al., 2000). In 2018, the WHO convened a workshop of experts in epidemiology, preclinical and clinical vaccine trials, mathematical modelling, and regulatory decision making to determine best practices for developing and testing plague vaccines. The WHO also developed a Target Product Profile to guide vaccine development.

Plague vaccines have a major limitation in that protection from plague is delayed after immunisation for a minimum of at least 1 week (Anisimov and Amoako, 2006). This time may be vital in prevention given the short incubation period of plague and its lethal nature (Anisimov and Amoako, 2006). Because of this limitation, antibiotics are used in early prophylaxis and as a treatment of plague. Indeed, the World WHO Expert Committee on Plague saw the use of antimicrobials as the foundation of plague treatment (WECo, 1970). However, bacterial resistance to antibiotics is becoming a global challenge. Two strains of *Yersinia pestis* have been identified as antibiotic resistant (Galimand et al., 2006; Ditchburn and Hodgkins, 2019). Given the rising level of antibiotic resistance to bacterial infectious diseases, it is important that alternative prevention strategies are developed, including effective and safe vaccines.

In this systematic literature review we sought to identify and evaluate any evidence that compared the efficacy, safety, and immunogenicity of any vaccines for bubonic and pneumonic plague compared to placebo, control vaccines, or no intervention in healthy adults. The purpose of this study is to provide a comprehensive, up to date review of the vaccines currently available for plague prevention.

## 2 Material and methods

### 2.1 Design

The protocol for this systematic review was developed in accordance with guidance from the Cochrane Handbook (Higgins et al., 2019) and the Preferred Reporting Items for Systematic reviews and Meta-Analyses (PRISMA) protocols (Shamseer et al., 2015). All team members approved the final protocol. The findings of this systematic review are reported in accordance with the PRISMA guidelines (Moher et al., 2009).

### 2.2 Literature search

A comprehensive search strategy was developed using the Peer Review of Electronic Search Strategies (PRESS) checklist (McGowan et al., 2016). The Cochrane Highly Sensitive Search filter for RCTs was used (Higgins et al., 2019). Trials were identified through systematic searches of MEDLINE, Embase, and the Cochrane Library from database inception to July 2022. The preliminary search strategy developed for Embase was adapted for use in other databases (Appendix 1). No date or language limitations were used for the electronic database searches. In addition to the electronic databases, the websites of the Global Summit on Infectious Diseases and Infectious Diseases: Control and Prevention were searched for relevant conference abstracts from 2018 to 2022, as these conferences are not indexed in Embase. Reference lists of relevant systematic reviews were also checked for additional studies. Additionally, trial registries, ClinicalTrials.gov, and the WHO International Clinical Trials Registry Platform (ICTRP) were searched for ongoing trials or completed trials in July 2022.

### 2.3 Selection criteria

The systematic review eligibility criteria were defined according to the Population, Interventions, Comparators, and Outcomes (PICO) approach (Higgins et al., 2019) with no limitations based on geography or date. We included RCTs that had any follow-up periods which examined plague vaccines in healthy adults and children to determine the efficacy, safety, and immunogenicity of the vaccines in the prevention of both the bubonic and pneumonic plague. Interventions considered were live, attenuated, or killed vaccines or fractions thereof administered by any route and given at any dosage. Comparators were no intervention, placebo, or control vaccine (e.g. vaccines against other diseases). Outcomes included efficacy outcomes (number of cases avoided by vaccination [plague in the intervention or control arm]), safety outcomes (adverse events [AEs] including the number and severity), and immunologic outcomes (rise in antibody titre).

### 2.4 Screening process

Titles and abstracts of the potential studies identified from the literature searches were independently screened for inclusion by two reviewers using the prespecified inclusion and exclusion criteria. Disagreements between reviewers were resolved by consensus, and a third reviewer was consulted if consensus could not be reached. The full texts of articles included at the title and abstract screen were then reviewed by two independent reviewers. Disagreements were resolved by consensus or a third reviewer where discrepancies persisted.

### 2.5 Data items and abstraction process

A data extraction form was created for study characteristics and outcome data. This form was piloted before full extraction began. Data were extracted by one author and independently quality checked by a second author.

### 2.6 Risk of bias assessment

One review author assessed the risk of bias for each study using the criteria outlined in the Cochrane Handbook (Higgins et al., 2019). A second author independently quality checked the assessments. Any disagreements were discussed and, if a consensus could not be reached, were resolved by discussion with a third reviewer. Risk of bias was assessed using the following domains: random sequence generation, allocation concealment, blinding of participants and personnel, blinding of outcome assessment, incomplete outcome data, selective outcome reporting, and other bias. Each potential source of bias was classified as unclear, high, or low.

## 3 Results

### 3.1 Literature search

**Fig. 1:**
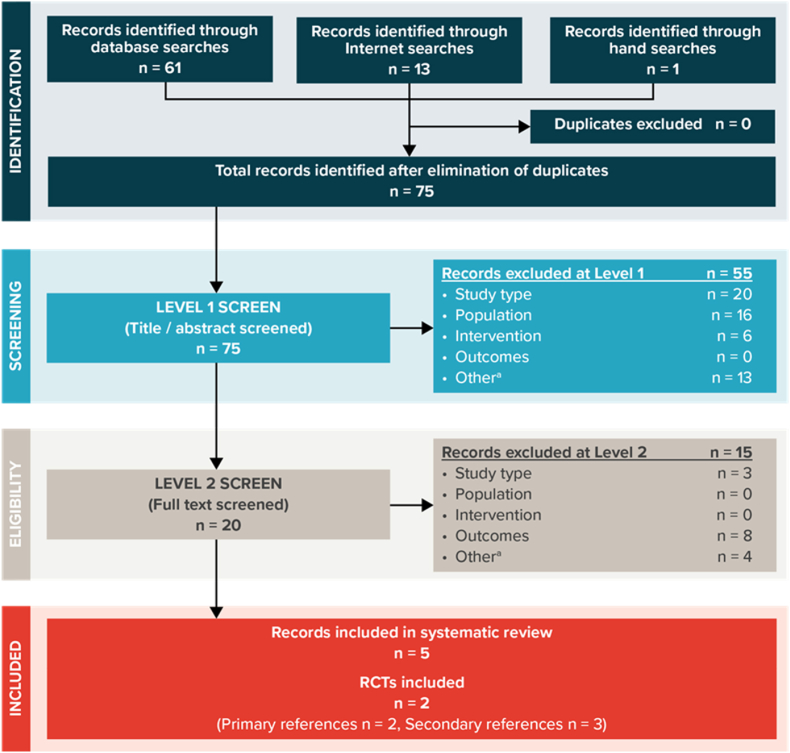
PRISMA Flow Diagram of Study Inclusion and Exclusion PRISMA = Preferred Reporting Items for Systematic reviews and Meta-Analyses; RCT = randomised controlled trial.

The search strategy yielded a total of 75 titles and abstracts to screen after duplicates had been removed (Fig. 1). Following screening of the titles and abstracts, 55 articles were excluded. An additional 15 articles were removed after reviewing full texts for the remaining 20 articles, which left 5 publications relating to 2 RCTs suitable for inclusion. All 5 articles were reported in English. No additional relevant completed trials were identified by examining the reference list of relevant systematic reviews or by searching conference abstracts. Nine RCTs were registered in the trial registries that investigated a vaccine for the prevention of plague. Six were marked as completed and one was marked as active but not recruiting. However, only 2 trials were found to have results available. These 2 trial records have been included as secondary publications for the 2 RCTs identified in the electronic database searches.

### 3.2 Study and patient characteristics

Chu et al. (2016) was a 2-arm trial that included a plague vaccine comprised of faction 1 (F1) and recombinant virulence (rV) antigens and aluminium adjuvant and compared 15 mcg of the recombinant subunit plague vaccine to 30 mcg of the plague vaccine. The vaccines were given in 2 doses 28 days apart. Frey et al. (2017) had 5 arms to their trial. Four arms were the subunit plague vaccine in which each arm received a different dosage of the Flagellin/F1/V vaccine. Doses were 1 mcg, 3 mcg, 6 mcg, and 10 mcg and were given by intramuscular injection on day 0 and after 28 days. The placebo arm was given a saline solution in the same volume as the plague vaccine in the respective group. Frey et al. (2017) was conducted in the United States and published in 2017. Chu et al. (2016) was conducted in China and published in 2016. Both RCTs used a parallel-group design with double blinding. Chu et al. (2016) was a phase 2a trial, while Frey et al. (2017) was phase 1. No phase 3 or 4 studies were identified.

Table 1 provides the characteristics of these trials. The duration of the trials varied considerably, with Chu et al. (2016) being only 12 weeks and Frey et al. (2017) lasting 13 months. Participant characteristics are presented in Table 2. The trials included healthy adults aged between 18 and 55 years old (Chu et al., 2016) and between 18 and ≤ 45 years old (Frey et al., 2017). For both trials, the percentage of male participants was similar (44.6% for Chu et al. (2016) and 52% for Frey et al. (2017)). Chu et al. (2016) had the largest sample size of the two trials, with 240 participants, compared to the 60 participants of Frey et al. (2017).

**Table 1:** Trial characteristics.

Study	Trial registry identifier	Study design	Study location	Study length	Outcomes reported
Chu et al. (2016)	NCT02596308	Phase 2a RCT	China	3 months	Immunogenicity Safety
Frey et al. (2017)	NCT01381744	Phase 1 RCT	US	13 months	Immunogenicity Safety

RCT = randomised controlled trial; US = United States.

**Table 2:** Patient characteristics.

Study	Health status	Study N	% male	Age, y	Trial arms
Chu et al. (2016)	Healthy adults	240	44.6	15-mcg Group: 43.12 30-mcg Group: 41.86	Plague vaccine 15 mcg (2 doses given at 28-day interval between doses) Plague vaccine 30 mcg (2 doses given at 28-day interval between doses)
Frey et al. (2017)	Healthy adults	60	52	Overall: 30.8	Flagellin/F1/V 1 mg (Intramuscular injection of 1 mcg/dose on days 0 and 20. Delivered in a volume of 100 mL) Flagellin/F1/V 3 mg (Intramuscular injection of 3 mcg/dose on days 0 and 28. Delivered in a volume of 300 mL [1 mcg/100 mL]) Flagellin/F1/V 6 mg (Intramuscular injection of 6 mcg/dose on days 0 and 28. Delivered in a volume of 600 mL [1 mcg/100 mL]) Flagellin/F1/V 10 mg (Intramuscular injection of 10 mcg/dose on days 0 and 28. Delivered in a volume of 100 mL) Placebo (The volume of placebo given was the same volume as the vaccine in the respective dose group)

### 3.3 Risk of bias results

**Fig. 2:**
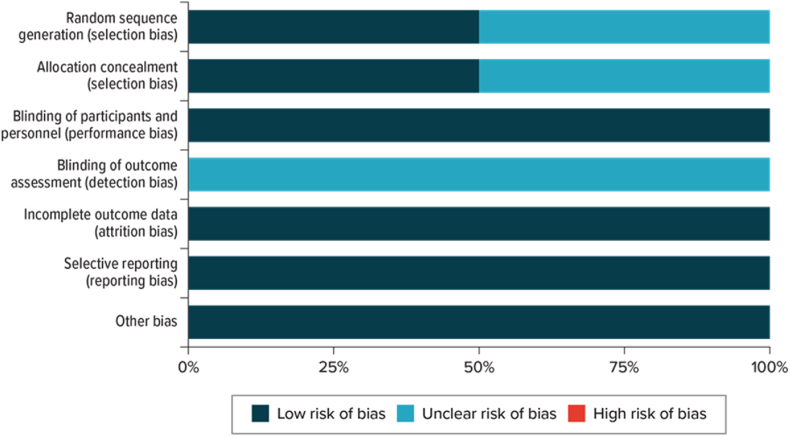
Risk of bias summary.

**Fig. 3:**
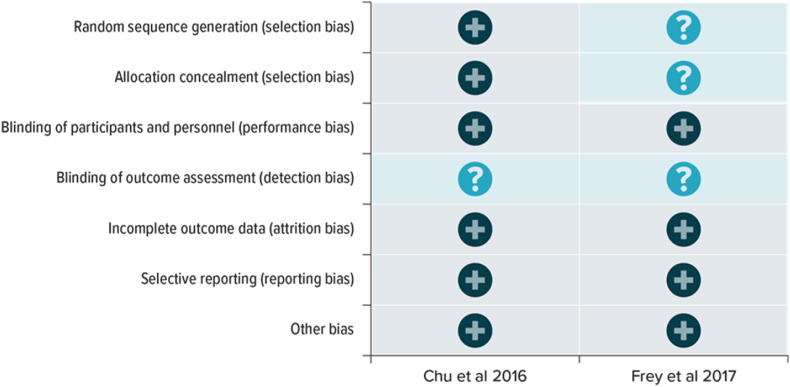
Risk of bias graph.

Both Chu et al. (2016) and Frey et al. (2017) had at least one domain classified as unclear risk of bias (Fig. 2, Fig. 3). Both studies had an unclear risk of detection bias due to inadequate reporting of the blinding of outcome assessment. Chu et al. (2016) was assessed as having a low risk of selection bias, performance bias, attrition bias, and reporting bias. Frey et al. (2017) was assessed as having a low risk of performance bias, attrition bias, and reporting bias. Additionally, Frey et al. (2017) had an unclear risk for selection bias and detection bias due to the inadequate reporting of random sequence generation, allocation concealment, and blinding of outcome assessment.

#### 3.3.1 Efficacy results

No data were identified as to the number of cases avoided by vaccination for either of the plague vaccines. However, both identified trials were at early phases (phase 1 and phase 2a), so safety and immunogenicity were the primary outcomes.

#### 3.3.2 Safety results

Both Chu et al. (2016) and Frey et al. (2017) reported AEs. However, the timepoints in which AE data were taken differed between the trials. Chu et al. (2016) reported solicited AEs within 7 days after vaccination, unsolicited AEs within 28 days after vaccination, and Serious AEs for 56 days and between day 56 and 6 months (Table 3). Frey et al. (2017) reported data on AEs 14 and 28 days after each dose of the vaccination.

**Table 3:** Adverse events reported in Chu et al. (2016).

AE	Grade 1		Grade 2		Grade 3	
	**15 mcg (n=119)**	**30 mcg (n=120)**	**15 mcg (n=119)**	**30 mcg (n=120)**	**15 mcg (n=119)**	**30 mcg (n=120)**
Solicited local AE within 7 days of vaccination						
Erythema/redness	6 (5.4%)	6 (5%)	2 (1.7%)	1 (0.8%)	1 (0.8%)	0 (0%)
Pain	29 (24.4%)	45 (37.5%)	2 (1.7%)	3 (2.5%)	0 (0%)	0 (0%)
Induration	1 (0.8%)	3 (2.5%)	1 (0.8%)	0 (0%)	0 (0%)	0 (0%)
Swelling	1 (0.8%)	1 (0.8%)	2 (1.7%)	4 (3.3%)	0 (0%)	0 (0%)
Pruritus	1 (0.8%)	0 (0%)	0 (0%)	0 (0%)	0 (0%)	0 (0%)
Solicited systemic AEs within 7 days of vaccination						
Fever	7 (5.9%)	10 (8.3%)	0 (0%)	0 (0%)	0 (0%)	0 (0%)
Muscle pain	2 (1.7%)	3 (2.5%)	1 (0.8%)	0 (0.%)	0 (0%)	0 (0%)
Fatigue	1 (0.8%)	0 (0%)	0 (0%)	0 (0%)	0 (0%)	0 (0%)
Headache	1 (0.8%)	3 (2.5%)	0 (0%)	0 (0%)	0 (0%)	0 (0%)
Unsolicited AEs within 28 days of vaccination	2 (1.7%)	4 (3.3%)	2 (1.7%)	0 (0%)	0 (0%)	1 (0.8%)

AE = adverse event.

In Chu et al. (2016), both doses of the vaccine given in two injections were well tolerated at all timepoints. The occurrence of both solicited and unsolicited AEs was similar between dose groups and, for both groups, most AEs were lower than grade 3. No serious AEs were reported between day 56 to 6 months, and only 1 serious AE was observed between month 6 and month 12, but this was not related to vaccination (Hu et al., 2018).

Frey et al. (2017) collected AE data at 14 and 28 days after each dose of the vaccination. No severe solicited AEs were reported, and most reported AEs were mild. Table 4 shows the solicited systemic and local reactions during the reporting period of the trial. Twenty-two participants reported unsolicited AEs, with a patient in the 1-mcg group experiencing a severe AE. Nine participants reported a non-serious AE that was associated with the vaccine. No serious adverse reactions related to the vaccine were reported.

**Table 4:** Adverse events reported in Frey et al. (2017).

AEs	Flagellin/F1/V				Placebo (n = 8)
	1 mg (n = 10)	3 mg (n = 10)	6 mg (n = 10)	10 mg (n = 10)	
Solicited systemic AEs	7 (70%)	7 (70%)	8 (80%)	5 (50%)	6 (75%)
Solicited local AEs	7 (70%)	10 (100%)	9 (90%)	10 (100%)	5 (62%)

AE = adverse event.

#### 3.3.3 Immunogenicity results

Chu et al. (2016) reported F1 and rV antigen subunit plague vaccine immunogenicity as a geometric mean titre (GMT) at both the baseline and post-vaccination for both studied doses (Chu et al., 2016; Hu et al., 2018). Geometric mean fold increase and seroconversion at day 28 and 56 were also reported. For the F1 antigen, seroconversion was defined as a 4-fold increase in antibodies after vaccination compared to pre-vaccination, while for the rV antigen seroconversion was defined as an rV antibody titre of at least 1:320 in initially seronegative participants and as a 4-fold increase in titre between pre-immunisation and post-vaccination. Results are shown in Table 5.

**Table 5:** Immunogenicity of the F1 and rV antibodies (Chu et al., 2016; Hu et al., 2018).

	15 mcg			30 mcg		
	GMT (95%CI)	Seroconversion rate	GMFI (95% CI)	GMT (95%CI)	Seroconversion rate	GMFI (95% CI)
F1 antibody						
Baseline	1.03 (0.99–1.07)	NA	NA	1.00 (1.00–1.00)	NA	NA
Day 28	13.88 (10.21–18.87)	78.99%	13.48 (9.97–18.24)	22.10 (16.31–29.91)	84.03%	22.10 (16.32–29.91)
Day 56	47.55 (36.29–62.31)	93.28%	46.19 (35.24–60.52)	90.53 (72.33–113.31)	96.64%	90.53 (72.33–113.31)
Month 6	165.31 (134.00–196.60)	100%	NR	270.33 (219.50–321.20)	100%	NR
Month 12	140.10 (93.94–186.30)	99.16%	NR	198.80 (152.30–245.20)	100%	NR
rV IgG antibody						
Baseline	11.07 (10.25–11.97)	NA	NA	10.66 (10.05–11.31)	NA	NA
Day 28	395.81 (278.12–563.30)	54.62%	35.74 (25.36–50.38)	551.71 (406.51–748.76)	71.43%	51.75 (38.12–70.25)
Day 56	2457.70 (2070.06–2917.93)	99.16%	221.94 (186.31–264.38)	2761.40 (2332.76–3268.79)	99.16%	259.00 (218.94–306.39)
Month 6	452.50 (325.80–579.20)	42.02%	NR	728.50 (358.10–1099.00)	49.15%	NR
Month 12	225.60 (123.30–347.90)	16.81%	NR	323.50 (204.90–442.10)	27.12%	NR

CI = confidence interval; F1 = faction 1; GMFI = geometric mean fold increase; GMT = geometric mean titre; IgG = immunoglobulin G; NR = not reported; rV = recombinant virulence.

For both vaccine doses, antibodies to the F1 antigen were increased from baseline to all timepoints. However, GMTs were higher at all timepoints post-vaccination for the 30-mcg group compared to the 15-mcg group (day 28: *P* = 0.0338, day 56: *P* = 0.0004, month 6: *P* < 0.0001, month 12: *P* < 0.0010). For the F1 antigen, the trial showed similar seroconversion rates for both doses across all time points. Seroconversion rates ranged from 78.99% to 84.03% at 28 days to 99.16% to 100% at 12 months. For the rV antigen, a decrease in antibodies between baseline and all timepoints post-vaccination was found for both doses of plague vaccine. GMTs were similar between both vaccine doses at 56 days (*P* = 0.3387). However, GMTs were numerically higher both 6 and 12 months post-vaccination for the 30-mcg group compared with the 15-mcg group. At 56 days post-vaccination, the seroconversion rate was the same between dosing groups (99.16% vs. 99.16%; *P* = 1.0000). However, the seroconversion rates decreased by almost 50% after 6 months compared to day 56 and decreased further after 12 months. For both the 15-mcg and 30-mcg formulations, 2 doses of the vaccine were found to be more immunogenic than just 1 dose.

Frey et al. (2017) reported peak immunoglobulin G (IgG) enzyme-linked immunosorbent assay (ELISA) antibody titres to the F1 antigen and the V antigen, but not baseline values. Peak titres were defined as the single maximum titre among all available measurements before first vaccination to 180 days after the 2 vaccinations. Results are shown in Table 6. Frey et al. (2017) found the peak ELISA IgG antibody titres after 2 doses of 10 mcg of vaccine to be 260.0 (102.6–659.0) for the F1 antigen and 983.6 (317.3–3048.8) for the V antigen. The titres were similar in the 6-mcg dose group. Antibody titres were low among the placebo group and the 1-mcg and 3-mcg dose groups. No participants in the placebo group had a 4-fold increase in F1 antigens, while 10% of the 1-mcg group, 20% of the 3-mcg group, 55.6% of the 6-mcg group, and 60% of the 10-mcg group showed a 4-fold increase. This increase was significant compared to placebo for the 6-mcg group (*P* = 0.029) and the 10-mcg group (*P* = 0.0130). No participants in the placebo group demonstrated a 4-fold increase in V antigen antibody titre, compared to 20% of the 1-mcg group, 30% of the 3-mcg group, 88.9% of the 6-mcg group, and 70% of the 10-mcg group. Compared to placebo, the four-fold increase in V antigen antibody titre was significant for the 6-mcg group (*P* < 0.001) and the 10-mcg group (*P* = 0.004).

**Table 6:** ELISA Titre Immunoglobulin G Responses Post Vaccination in Frey et al. (2017).

	Flagellin/F1/V				
	1 μg	3 μg	6 μg	10 μg	Placebo
F1 antigen					
GMT (95% CI)	71.8 (46.8–110.2)	89.5 (49.5–161.6)	273.1 (167.5–445.4)	260.0 (102.6–659.0)	61.7 (37.6–101.2)
GMFR (95%CI)	1.4 (0.9–2.2)	1.8 (1.0–3.2)	4.5 (2.7–7.5)	5.2 (2.1–13.2)	1.0 (1.0–1.1)
Four-fold increase % (95% CI)	10.0 (0.3–44.5)	20.0 (2.5–55.6)	55.6 (21.2–86.3)	60.0 (26.2–87.8)	0.0 (0.0–36.9)
V antigen					
GMT (95% CI)	101.4 (56.5–181.8)	167.1 (72.5–385.2)	963.3 (641.5–1446.8)	983.6 (317.3–3048.8)	64.9 (43.3–97.3)
GMFR (95%CI)	2.0 (1.1–3.6)	3.3 (1.4–7.7)	14.3 (7.0–29.1)	19.7 (6.3–61.0)	1.1 (0.9–1.3)
Four-fold increase % (95% CI)	20.0 (2.5–55.6)	30.0 (6.7–65.2)	88.9 (51.8–99.7)	70.0 (34.8–93.3)	0.0 (0.0–36.9)

CI = confidence interval; GMFR = geometric mean fold rise; GMT = geometric mean titre.

## 4 Discussion

This review sought to assess the efficacy, safety, and immunogenicity of current vaccines to prevent the plague. Evidence from clinical trials was limited, with only 2 trials identified, both of which were early phases (phase 1 and phase 2a). Neither reported efficacy outcomes, and the safety and immunogenicity endpoints differed between the 2 studies. One trial was of reasonable quality, and the other trial had several risks due to inadequate reporting of key methodological components.

With regards to safety, both vaccines were well tolerated and safe, with primarily mild to moderate AEs recorded. However, the included trials reported only short-term data, so no conclusions about the long-term side effects of these vaccines can be made. Immune responses were reported for both subunit plague vaccines. Chu et al. (2016) reported that a recombinant subunit plague vaccine with F1 and rV antigens had robust immunogenicity for up to 12 months after vaccination. It was also found that 2 doses of the F1/rV antigen plague vaccine were more immunogenic than only 1 dose for both the 30-mcg and 15-mcg formulations. However, the 30-mcg dose was found to be, on the whole, more immunogenic than the 15-mcg dose, suggesting that this would be the preferred dose for any further development. Similarly, Frey et al. (2017) reported a dose-dependent response to a subunit vaccine with F1 and V antigens and Flagellin, with immune responses increasing as vaccine doses increased from 1 mcg to 10 mcg. Nonetheless, for both trials, the observation time for immunity was short (i.e., the durability of immunity cannot be assessed).

The lack of clinical trials on plague vaccines found by this review indicates a significant evidence gap in plague vaccine research and the need for additional, rigorous long-term studies.

### 4.1 Strengths and limitations

A major strength of this review is the methodology by which it was performed. A comprehensive literature search of journal articles, clinical trial registries, and conference proceedings, including those not indexed in electronic databases, was conducted. No language restrictions were placed on the search, reducing selection bias. This review also complied to PRISMA guidance, and all outcomes were prespecified in the protocol. Furthermore, the review included independent study identification, selection, and extraction by 2 reviewers to reduce bias. However, there are some limitations to this review. Although searches were conducted in ClinicalTrials.gov and the ICTRP, it is possible that additional unpublished trials exist, leading to publication bias. However, the limited number of studies included in the review did not allow for an assessment of publication bias. Additionally, the planned analyses were not conducted due to the limited number of trials available and heterogeneity between these trials.

### 4.2 Comparison with other studies

Other systematic literature reviews investigating the efficacy and safety of vaccines for the prevention of plague are lacking. Only 1 other systematic review was identified (Jefferson et al., 2011). This review was originally conducted in 1998 and updated in 2006, 2009, and 2011; however, no RCTs of vaccines for the prevention of plague were identified. Jefferson et al. (2000) concluded that there was not enough evidence from RCTs to investigate the efficacy or safety of any plague vaccines nor compare their relative effectiveness. However, evidence from observational studies indicated that killed types of plague vaccines may be more effective and have better tolerability than attenuated vaccines (Jefferson et al., 2000). Furthermore, only 6 RCTs have been registered in ClinicalTrials.gov that investigate a vaccine for the prevention of plague. While all 6 RCTs are marked as completed, only 2 have results available (Chu et al., 2016; Frey et al., 2017) and only 1 reported long-term immunogenicity (up to 12 months after vaccination) (Hu et al., 2018).

### 4.3 Policy implications

This systematic review found no evidence for the efficacy of plague vaccines and found limited evidence for the safety and immunogenicity of these vaccines. Only 2 RCTs were identified, and both were early phase trials. Given the lack of evidence identified, conclusions on the efficacy, safety, and immunogenicity of any plague vaccine cannot be determined at this time. However, this systematic review does highlight the need for well-designed, long-term, large RCTs assessing efficacy, safety, and immunogenicity of vaccines designed to prevent plague. Future trials should include outcomes for plague incidence, mortality related to the vaccines, and AEs related to the administration of the vaccine. Furthermore, future trials should be long-term so that the durability of immunity can be assessed and long-term side effects of the vaccines can be identified.

The limitations found in these clinical trials are in line with those identified by the WHO report in 2018 (World Health Organization, 2018). As stated in the WHO report, efficacy outcomes for plague vaccines can be difficult to measure, and we did not identify any studies that included efficacy outcomes. The observation time in these studies, however, was short. Future trials would likely need longer observational periods in order to accurately determine the efficacy of plague vaccines. Because of the limited information reported by these trials, it is difficult to say whether the trials presented in Chu et al. and Frey et al. were conducted in line with WHO recommendations.

## 5 Conclusions

At present, there is not enough evidence to evaluate the efficacy, safety, and immunogenicity of any vaccines to prevent either the bubonic or pneumonic plague. Furthermore, evidence as to the long-term effects of plague vaccines is scarce, with only 2 early phase, short-term trials identified.

## Ethics approval and consent to participate

Not applicable.

## Consent for publication

Not applicable.

## Availability of data and materials

The full dataset and statistical code are available from the corresponding author on reasonable request.

## Funding

This research did not receive any specific grant from funding agencies in the public, commercial, or not-for-profit sectors.

## CRediT authorship contribution statement

**Louise Hartley:** Conceptualization, Data curation, Methodology, Investigation, Formal analysis, Visualization, Project administration, Writing – original draft, Writing – review & editing. **Sydney Harold:** Validation, Investigation, Writing – review & editing. **Emma Hawe:** Methodology, Data curation, Supervision, Writing – review & editing.

## Declaration of competing interest

All authors have completed the ICMJE uniform disclosure form at www.icmje.org/coi_disclosure.pdf. LH, SH, and EH have no financial relationships with any organisations that might have an interest in the submitted work in the previous three years; and no other relationships or activities to declare that could appear to have influenced the submitted work.

## Figures and Tables

**Table:** 

Embase Literature Search Strategy:	
Search Number	Search Terms
**Population of interest**	
#1	'plague'/exp OR 'plague':ti,ab,de OR 'pneumonic plague'/exp OR 'pneumonic plague':ti,ab,de OR ‘pulmonic plague’:ti,ab,de OR 'bubonic plague'/exp OR 'bubonic plague':ti,ab,de OR ‘bacterial plague’:ti,ab,de OR ‘oriental plague’:ti,ab,de OR ‘yersinia pestis infection’:ti,ab,de OR ‘black death’:ti,ab,de OR ‘black plague’:ti,ab,de
**Intervention**	
#2	'plague vaccine'/exp OR 'plague vaccin*':ti,ab,de OR 'vaccine'/exp OR 'vaccin*':ti,ab,de
**Study Design**	
#3	'crossover procedure':de OR 'double-blind procedure':de OR 'randomized controlled trial':de OR 'single-blind procedure':de OR (random* OR factorial* OR crossover* OR cross NEXT/1 over* OR placebo* OR doubl* NEAR/1 blind* OR singl* NEAR/1 blind* OR assign* OR allocat* OR volunteer*):de,ab,ti
**Exclusion terms**	
#4	‘animal’/exp NOT ‘human’/exp
#5	comment*:ti OR ‘letter’:it OR ‘editorial’:it OR ‘case report’/exp OR ‘case stud*’:ti OR ‘case report*’:ti OR ‘case series’:ti OR ‘note’:it
**All relevant studies**	
#6	#1 AND #2 AND #3
#7	#6 NOT (#4 OR #5)

**Table:** 

PubMed Literature Search Strategy	
Search Number	Search Terms
**Population of interest**	
#1	'plague'[Mesh] OR 'plague'[Text] OR 'pneumonic plague'[Text] OR ‘pulmonic plague’ [Text] OR 'bubonic plague' [Text] OR ‘bacterial plague’ [Text] OR ‘oriental plague’ [Text] OR ‘yersinia pestis infection’[Text] OR ‘Black death’ [Text] OR ‘Black plague’[Text]
**Intervention**	
#2	'plague vaccine'[Mesh] OR 'plague vaccine'[Text] OR ‘Plague vaccines’[Text] OR ‘plague vaccination’[Text] OR vaccines[Mesh] OR vaccine[Text] OR vaccines[Text] OR vaccination[Text]
**Study Design**	
#3	randomized controlled trial[pt] OR controlled clinical trial[pt] OR randomized[title/abstract] OR placebo[title/abstract] OR clinical trials as topic[mesh:noexp] OR randomly[title/abstract] OR trial[title]
**Exclusion terms**	
#4	“Animals”[Mesh] NOT “Humans”[Mesh]
#5	“Comment”[Publication Type] OR “Letter”[Publication Type] OR “Editorial”[Publication Type] OR “Case Reports”[Publication Type] OR case stud*[Title] OR case report*[Title] OR “case series”[Title] OR case histor*[Title]
**All relevant studies**	
#6	#1 AND #2 AND #3
#7	#6 NOT (#4 OR #5)

**Table:** 

Cochrane Library Literature Search Strategy	
Search Number	Search Terms
Disease	
#1	MeSH descriptor: [Plague] explode all trees
#2	(“plague”):ti,ab,kw
#3	(“pneumonic plague”):ti,ab,kw
#4	(“pulmonic plague”):ti,ab,kw
#5	(bubonic plague):ti,ab,kw
#6	(bacterial plague):ti,ab,kw
#7	(oriental plague):ti,ab,kw
#8	(yersinia pestis infection):ti,ab,kw
#9	(black death):ti,ab,kw
#10	(“black plague”):ti,ab,kw
#11	#1 OR #2 OR #3 OR #4 OR #5 OR #6 OR #7 OR #8 OR #9 OR #10
**Intervention**	
#12	MeSH descriptor:[Plague Vaccine] explode all trees
#13	(“plague vaccine”):ti,ab,kw
#14	(Plague Vaccines):ti,ab,kw
#15	(Plague Vaccinations):ti,ab,kw
#16	MeSH descriptor:[Vaccines] explode all trees
#17	MeSH descriptor:[Vaccination] explode all trees
#18	(“vaccine”):ti,ab,kw
#19	(“vaccination”):ti,ab,kw
#20	#12 OR #13 OR #14 OR #15 OR #16 OR #17 OR #18 OR #19
**RCT**	
#21	("randomized controlled trial" OR "controlled clinical trial"):pt
#22	(randomized OR placebo OR randomly OR trial OR groups):ti,ab,kw
#23	#21 OR #22
**Exclusion terms**	
#24	MeSH descriptor: [Animals] explode all trees
#25	MeSH descriptor: [Humans] explode all trees
#26	#24 NOT #25
#27	("Comment" OR "Letter" OR "Editorial" OR "Case Reports" OR "Clinical Trial Phase I"):pt
#28	(case NEXT stud* OR case NEXT report* OR "case series" OR case NEXT histor*):ti
#29	#27 OR #28
**Total**	
#30	#11 AND #20 AND #23
#31	#30 NOT (#26 OR #29)

## Data Availability

No data were used for the research described in the article.

## Abbreviations

AE: adverse event
ELISA: enzyme-linked immunosorbent assay
F1: faction 1
GMFI: geometric mean fold increase
GMT: geometric mean titer
ICTRP: International Clinical Trials Registry Platform
IgG: immunoglobulin G
PICO: Population, Interventions, Comparators, and Outcomes
PRESS: Peer Review of Electronic Search Strategies
PRISMA: Preferred Reporting Items for Systematic Reviews and Meta-Analyses
RCT: randomised controlled trial
rV: recombinant virulence
US: United States
WHO: World Health Organisation
